# 1-[3-(4-Chloro­phen­yl)isoquinolin-1-yl]-3,5-diphenyl-1*H*-pyrazole

**DOI:** 10.1107/S1600536809055718

**Published:** 2010-01-16

**Authors:** F. Nawaz Khan, P. Manivel, V. Krishnakumar, Venkatesha R. Hathwar, Seik Weng Ng

**Affiliations:** a Chemistry Division, School of Science and Humanities, VIT University, Vellore 632014, Tamil Nadu, India; b Chemistry Division, School of Advanced Sciences, VIT University, Vellore 632014, Tamil Nadu, India; c Solid State and Structural Chemistry Unit, Indian Institute of Science, Bangalore 560012, Karnataka, India; d Department of Chemistry, University of Malaya, 50603 Kuala Lumpur, Malaysia

## Abstract

The title compound, C_30_H_20_ClN_3_, is composed of a diaryl-substituted pyrazole ring connected to an aryl-substituted isoquinoline ring system with a dihedral angle of 65.1 (1)° between the pyrazole ring and the isoquinoline ring system. The 3-phenyl and 4-phenyl substitutents are twisted by 8.1 (1) and 43.0 (1)°, respectively, with respect to the pyrazole ring. The chloro­phenyl ring and the isoquinoline ring system are twisted by 21.2 (1)° with respect to each other.

## Related literature

For medicinal applications of hydrazine derivatives, see: Broadhurst *et al.* (2001 ▶).
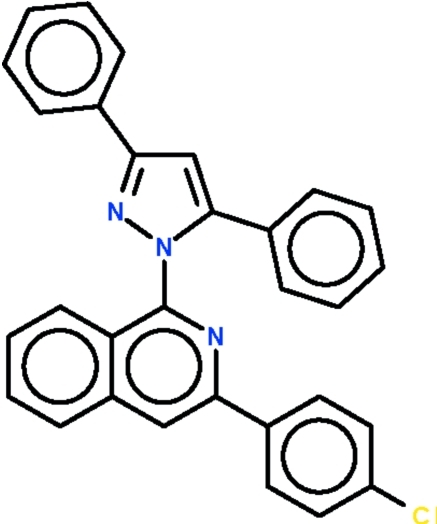


## Experimental

### 

#### Crystal data

C_30_H_20_ClN_3_
*M* *_r_* = 457.94
Monoclinic, 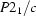

*a* = 23.7864 (15) Å
*b* = 11.7101 (8) Å
*c* = 8.3602 (5) Å
β = 96.738 (1)°
*V* = 2312.6 (3) Å^3^
*Z* = 4
Mo *K*α radiation
μ = 0.19 mm^−1^
*T* = 290 K
0.20 × 0.16 × 0.04 mm

#### Data collection

Bruker SMART area-detector diffractometer
Absorption correction: multi-scan (*SADABS*; Sheldrick, 1996 ▶) *T*_min_ = 0.963, *T*_max_ = 0.993
17230 measured reflections
4392 independent reflections
2890 reflections with *I* > 2σ(*I*)
*R*_int_ = 0.039

#### Refinement

*R*[*F* ^2^ > 2σ(*F* ^2^)] = 0.050
*wR*(*F* ^2^) = 0.126
*S* = 1.02
4392 reflections
307 parameters
H-atom parameters constrained
Δρ_max_ = 0.16 e Å^−3^
Δρ_min_ = −0.19 e Å^−3^

### 

Data collection: *SMART* (Bruker, 2004 ▶); cell refinement: *SAINT* (Bruker, 2004 ▶); data reduction: *SAINT*; program(s) used to solve structure: *SHELXS97* (Sheldrick, 2008 ▶); program(s) used to refine structure: *SHELXL97* (Sheldrick, 2008 ▶); molecular graphics: *X-SEED* (Barbour, 2001 ▶); software used to prepare material for publication: *publCIF* (Westrip, 2010 ▶).

## Supplementary Material

Crystal structure: contains datablocks global, I. DOI: 10.1107/S1600536809055718/bt5160sup1.cif

Structure factors: contains datablocks I. DOI: 10.1107/S1600536809055718/bt5160Isup2.hkl

Additional supplementary materials:  crystallographic information; 3D view; checkCIF report

## Figures and Tables

**Fig. 1.**
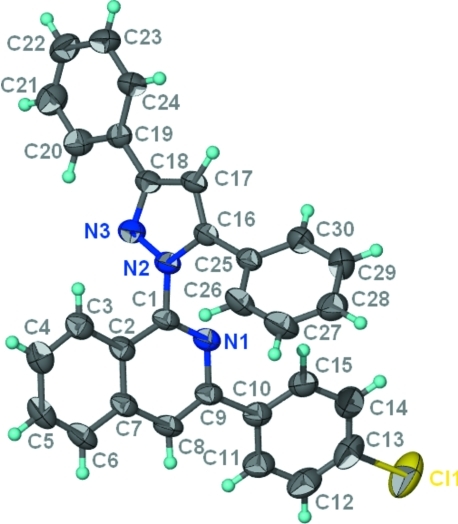
Anisotropic displacement ellipsoid plot (Barbour, 2001) of C30H20ClN3 at the 50% probability level; hydrogen atoms are drawn as spheres of arbitrary radius.

## supplementary crystallographic information

### Experimental

1-[3-(4-Chlorophenyl)isoquinolin-1-yl]hydrazine (2.69 g, 10 mmol) and 1,3-diphenylpropane-1,3-dione (2.24 g, 10 mmol) were dissolved in ethanol (30 ml). The solution was heated for 12 h under a nitrogen atmosphere. The reaction was quenched with water; the compound was extracted with ethyl acetate. The ethyl acetate phase was washed with water, dried, concentrated and purified by column chromatography to yield a white powder. Crystals were were obtained upon recrystallization from dichloromethane.

### Refinement

Hydrogen atoms were placed in calculated positions (C–H 0.93 Å) and were included in the refinement in the riding model approximation, with *U*(H) set to 1.2*U*_eq_(C).

### Crystal data

**Table:**

C_30_H_20_ClN_3_	*F*(000) = 952
*M**_r_* = 457.94	*D*_x_ = 1.315 Mg m^−^^3^
Monoclinic, *P*2_1_/*c*	Mo *K*α radiation, λ = 0.71073 Å
Hall symbol: -P 2ybc	Cell parameters from 3042 reflections
*a* = 23.7864 (15) Å	θ = 2.4–21.9°
*b* = 11.7101 (8) Å	µ = 0.19 mm^−^^1^
*c* = 8.3602 (5) Å	*T* = 290 K
β = 96.738 (1)°	Plate, colorless
*V* = 2312.6 (3) Å^3^	0.20 × 0.16 × 0.04 mm
*Z* = 4	

### Data collection

**Table:**

Bruker SMART area-detector diffractometer	4392 independent reflections
Radiation source: fine-focus sealed tube	2890 reflections with *I* > 2σ(*I*)
graphite	*R*_int_ = 0.039
φ and ω scans	θ_max_ = 25.7°, θ_min_ = 1.7°
Absorption correction: multi-scan (*SADABS*; Sheldrick, 1996)	*h* = −29→26
*T*_min_ = 0.963, *T*_max_ = 0.993	*k* = −14→14
17230 measured reflections	*l* = −10→10

### Refinement

**Table:**

Refinement on *F*^2^	Primary atom site location: structure-invariant direct methods
Least-squares matrix: full	Secondary atom site location: difference Fourier map
*R*[*F*^2^ > 2σ(*F*^2^)] = 0.050	Hydrogen site location: inferred from neighbouring sites
*wR*(*F*^2^) = 0.126	H-atom parameters constrained
*S* = 1.02	*w* = 1/[σ^2^(*F*_o_^2^) + (0.0562*P*)^2^ + 0.2618*P*] where *P* = (*F*_o_^2^ + 2*F*_c_^2^)/3
4392 reflections	(Δ/σ)_max_ = 0.001
307 parameters	Δρ_max_ = 0.16 e Å^−^^3^
0 restraints	Δρ_min_ = −0.19 e Å^−^^3^

### Fractional atomic coordinates and isotropic or equivalent isotropic displacement parameters (Å^2^)

**Table:**

	*x*	*y*	*z*	*U*_iso_*/*U*_eq_	
Cl1	0.50745 (3)	0.63807 (9)	1.25356 (11)	0.1273 (4)	
N1	0.26392 (7)	0.53740 (12)	0.79095 (19)	0.0492 (4)	
N2	0.19665 (7)	0.41903 (13)	0.65629 (19)	0.0491 (4)	
N3	0.14743 (7)	0.38380 (13)	0.70881 (19)	0.0528 (4)	
C1	0.21697 (9)	0.53202 (15)	0.6937 (2)	0.0464 (5)	
C2	0.18694 (9)	0.62628 (16)	0.6200 (2)	0.0474 (5)	
C3	0.13772 (9)	0.61722 (18)	0.5098 (2)	0.0575 (6)	
H3	0.1220	0.5459	0.4843	0.069*	
C4	0.11301 (10)	0.7131 (2)	0.4401 (3)	0.0649 (6)	
H4	0.0806	0.7067	0.3668	0.078*	
C5	0.13619 (11)	0.82097 (19)	0.4784 (3)	0.0657 (6)	
H5	0.1188	0.8856	0.4305	0.079*	
C6	0.18349 (10)	0.83272 (17)	0.5840 (3)	0.0604 (6)	
H6	0.1982	0.9051	0.6081	0.072*	
C7	0.21069 (9)	0.73527 (16)	0.6579 (2)	0.0495 (5)	
C8	0.26116 (9)	0.74042 (16)	0.7625 (2)	0.0545 (5)	
H8	0.2775	0.8111	0.7891	0.065*	
C9	0.28696 (9)	0.64306 (16)	0.8263 (2)	0.0486 (5)	
C10	0.34144 (9)	0.64280 (17)	0.9322 (2)	0.0530 (5)	
C11	0.36151 (10)	0.73945 (19)	1.0167 (3)	0.0661 (6)	
H11	0.3401	0.8061	1.0078	0.079*	
C12	0.41255 (12)	0.7383 (2)	1.1135 (3)	0.0797 (7)	
H12	0.4255	0.8038	1.1691	0.096*	
C13	0.44384 (10)	0.6405 (3)	1.1273 (3)	0.0770 (7)	
C14	0.42564 (10)	0.5433 (2)	1.0454 (3)	0.0756 (7)	
H14	0.4474	0.4772	1.0549	0.091*	
C15	0.37442 (10)	0.54535 (19)	0.9486 (3)	0.0638 (6)	
H15	0.3618	0.4796	0.8932	0.077*	
C16	0.22447 (8)	0.33297 (15)	0.5886 (2)	0.0457 (5)	
C17	0.19129 (9)	0.23868 (16)	0.5973 (2)	0.0503 (5)	
H17	0.1985	0.1658	0.5606	0.060*	
C18	0.14428 (8)	0.27279 (15)	0.6720 (2)	0.0469 (5)	
C19	0.09634 (8)	0.20395 (16)	0.7112 (2)	0.0487 (5)	
C20	0.05671 (10)	0.24761 (19)	0.8028 (3)	0.0621 (6)	
H20	0.0602	0.3226	0.8393	0.075*	
C21	0.01216 (10)	0.1822 (2)	0.8410 (3)	0.0750 (7)	
H21	−0.0137	0.2129	0.9044	0.090*	
C22	0.00553 (11)	0.0715 (2)	0.7861 (3)	0.0740 (7)	
H22	−0.0248	0.0275	0.8113	0.089*	
C23	0.04403 (11)	0.02741 (19)	0.6942 (3)	0.0742 (7)	
H23	0.0398	−0.0470	0.6558	0.089*	
C24	0.08906 (10)	0.09214 (17)	0.6579 (3)	0.0645 (6)	
H24	0.1152	0.0603	0.5963	0.077*	
C25	0.27834 (9)	0.34958 (15)	0.5214 (2)	0.0479 (5)	
C26	0.28817 (10)	0.44330 (17)	0.4282 (3)	0.0641 (6)	
H26	0.2597	0.4971	0.4039	0.077*	
C27	0.33964 (12)	0.45783 (19)	0.3709 (3)	0.0758 (7)	
H27	0.3457	0.5217	0.3090	0.091*	
C28	0.38175 (11)	0.3795 (2)	0.4041 (3)	0.0734 (7)	
H28	0.4167	0.3902	0.3669	0.088*	
C29	0.37204 (11)	0.2852 (2)	0.4925 (3)	0.0769 (7)	
H29	0.4004	0.2306	0.5139	0.092*	
C30	0.32081 (9)	0.26987 (18)	0.5502 (3)	0.0646 (6)	
H30	0.3148	0.2048	0.6096	0.078*	

### Atomic displacement parameters (Å^2^)

**Table:**

	*U*^11^	*U*^22^	*U*^33^	*U*^12^	*U*^13^	*U*^23^
Cl1	0.0696 (5)	0.1876 (10)	0.1191 (7)	−0.0076 (5)	−0.0121 (4)	−0.0404 (6)
N1	0.0585 (11)	0.0386 (9)	0.0517 (10)	−0.0025 (8)	0.0112 (9)	−0.0036 (7)
N2	0.0550 (11)	0.0364 (9)	0.0564 (10)	−0.0014 (8)	0.0090 (8)	−0.0034 (7)
N3	0.0581 (11)	0.0424 (10)	0.0585 (10)	−0.0037 (8)	0.0096 (9)	−0.0011 (8)
C1	0.0581 (13)	0.0349 (11)	0.0483 (11)	−0.0019 (9)	0.0153 (10)	−0.0056 (8)
C2	0.0565 (13)	0.0421 (11)	0.0463 (11)	0.0043 (10)	0.0169 (10)	−0.0021 (9)
C3	0.0660 (15)	0.0528 (13)	0.0557 (12)	0.0036 (11)	0.0153 (11)	−0.0019 (10)
C4	0.0685 (16)	0.0687 (16)	0.0585 (13)	0.0150 (13)	0.0119 (11)	0.0035 (11)
C5	0.0850 (18)	0.0551 (14)	0.0603 (14)	0.0241 (13)	0.0230 (13)	0.0083 (11)
C6	0.0831 (17)	0.0393 (12)	0.0626 (13)	0.0115 (11)	0.0248 (13)	0.0002 (10)
C7	0.0627 (14)	0.0396 (11)	0.0497 (11)	0.0039 (10)	0.0221 (10)	−0.0024 (9)
C8	0.0692 (15)	0.0357 (11)	0.0620 (13)	−0.0058 (10)	0.0225 (12)	−0.0077 (9)
C9	0.0576 (13)	0.0406 (12)	0.0509 (11)	−0.0055 (10)	0.0191 (10)	−0.0069 (9)
C10	0.0591 (14)	0.0510 (13)	0.0514 (12)	−0.0087 (10)	0.0171 (10)	−0.0048 (10)
C11	0.0729 (17)	0.0604 (14)	0.0668 (15)	−0.0103 (12)	0.0166 (13)	−0.0143 (11)
C12	0.0724 (18)	0.093 (2)	0.0762 (17)	−0.0232 (16)	0.0188 (14)	−0.0332 (14)
C13	0.0535 (15)	0.110 (2)	0.0684 (16)	−0.0136 (15)	0.0116 (12)	−0.0142 (15)
C14	0.0608 (17)	0.0819 (18)	0.0838 (17)	−0.0015 (13)	0.0074 (14)	−0.0025 (14)
C15	0.0633 (15)	0.0564 (14)	0.0724 (15)	−0.0081 (12)	0.0105 (12)	−0.0051 (11)
C16	0.0517 (12)	0.0366 (11)	0.0475 (11)	0.0026 (9)	0.0009 (9)	−0.0013 (8)
C17	0.0600 (14)	0.0337 (11)	0.0557 (12)	0.0010 (10)	0.0011 (10)	−0.0030 (9)
C18	0.0543 (13)	0.0386 (11)	0.0461 (11)	−0.0010 (9)	−0.0012 (9)	0.0008 (8)
C19	0.0505 (12)	0.0454 (12)	0.0479 (11)	−0.0014 (10)	−0.0037 (9)	0.0056 (9)
C20	0.0647 (15)	0.0594 (14)	0.0625 (14)	−0.0055 (12)	0.0082 (12)	−0.0038 (11)
C21	0.0671 (17)	0.0862 (19)	0.0739 (16)	−0.0064 (14)	0.0178 (13)	−0.0008 (14)
C22	0.0609 (16)	0.0766 (18)	0.0824 (17)	−0.0146 (14)	0.0000 (14)	0.0200 (14)
C23	0.0715 (17)	0.0490 (14)	0.1004 (19)	−0.0119 (13)	0.0028 (15)	0.0067 (13)
C24	0.0656 (15)	0.0432 (13)	0.0849 (16)	−0.0042 (11)	0.0090 (12)	−0.0001 (11)
C25	0.0546 (13)	0.0377 (11)	0.0503 (11)	0.0008 (9)	0.0019 (9)	−0.0062 (8)
C26	0.0801 (17)	0.0449 (12)	0.0722 (15)	0.0134 (11)	0.0285 (13)	0.0067 (10)
C27	0.100 (2)	0.0476 (14)	0.0877 (17)	0.0030 (14)	0.0450 (16)	0.0004 (12)
C28	0.0662 (16)	0.0680 (16)	0.0895 (18)	−0.0083 (14)	0.0239 (13)	−0.0155 (14)
C29	0.0578 (16)	0.0710 (17)	0.1009 (19)	0.0112 (13)	0.0049 (14)	0.0056 (15)
C30	0.0579 (15)	0.0520 (13)	0.0829 (16)	0.0016 (11)	0.0034 (12)	0.0113 (11)

### Geometric parameters (Å, °)

**Table:**

Cl1—C13	1.740 (3)	C14—H14	0.9300
N1—C1	1.304 (2)	C15—H15	0.9300
N1—C9	1.371 (2)	C16—C17	1.364 (2)
N2—N3	1.362 (2)	C16—C25	1.471 (3)
N2—C16	1.364 (2)	C17—C18	1.401 (3)
N2—C1	1.431 (2)	C17—H17	0.9300
N3—C18	1.336 (2)	C18—C19	1.465 (3)
C1—C2	1.416 (3)	C19—C20	1.380 (3)
C2—C3	1.406 (3)	C19—C24	1.387 (3)
C2—C7	1.416 (3)	C20—C21	1.375 (3)
C3—C4	1.366 (3)	C20—H20	0.9300
C3—H3	0.9300	C21—C22	1.378 (3)
C4—C5	1.400 (3)	C21—H21	0.9300
C4—H4	0.9300	C22—C23	1.364 (3)
C5—C6	1.353 (3)	C22—H22	0.9300
C5—H5	0.9300	C23—C24	1.375 (3)
C6—C7	1.417 (3)	C23—H23	0.9300
C6—H6	0.9300	C24—H24	0.9300
C7—C8	1.401 (3)	C25—C30	1.376 (3)
C8—C9	1.373 (3)	C25—C26	1.382 (3)
C8—H8	0.9300	C26—C27	1.377 (3)
C9—C10	1.481 (3)	C26—H26	0.9300
C10—C15	1.382 (3)	C27—C28	1.363 (3)
C10—C11	1.389 (3)	C27—H27	0.9300
C11—C12	1.378 (3)	C28—C29	1.363 (3)
C11—H11	0.9300	C28—H28	0.9300
C12—C13	1.364 (3)	C29—C30	1.374 (3)
C12—H12	0.9300	C29—H29	0.9300
C13—C14	1.372 (3)	C30—H30	0.9300
C14—C15	1.381 (3)		
			
C1—N1—C9	118.01 (16)	C14—C15—H15	119.2
N3—N2—C16	112.77 (15)	C10—C15—H15	119.2
N3—N2—C1	119.41 (15)	C17—C16—N2	105.32 (17)
C16—N2—C1	127.30 (17)	C17—C16—C25	131.50 (18)
C18—N3—N2	104.34 (15)	N2—C16—C25	123.17 (16)
N1—C1—C2	125.82 (17)	C16—C17—C18	106.85 (17)
N1—C1—N2	115.07 (16)	C16—C17—H17	126.6
C2—C1—N2	119.07 (18)	C18—C17—H17	126.6
C3—C2—C1	124.39 (18)	N3—C18—C17	110.72 (17)
C3—C2—C7	119.60 (18)	N3—C18—C19	120.53 (18)
C1—C2—C7	115.95 (19)	C17—C18—C19	128.74 (18)
C4—C3—C2	120.1 (2)	C20—C19—C24	117.39 (19)
C4—C3—H3	120.0	C20—C19—C18	121.37 (19)
C2—C3—H3	120.0	C24—C19—C18	121.24 (19)
C3—C4—C5	120.4 (2)	C21—C20—C19	121.2 (2)
C3—C4—H4	119.8	C21—C20—H20	119.4
C5—C4—H4	119.8	C19—C20—H20	119.4
C6—C5—C4	121.0 (2)	C20—C21—C22	120.5 (2)
C6—C5—H5	119.5	C20—C21—H21	119.7
C4—C5—H5	119.5	C22—C21—H21	119.7
C5—C6—C7	120.3 (2)	C23—C22—C21	119.1 (2)
C5—C6—H6	119.8	C23—C22—H22	120.5
C7—C6—H6	119.8	C21—C22—H22	120.5
C8—C7—C2	117.93 (17)	C22—C23—C24	120.5 (2)
C8—C7—C6	123.46 (19)	C22—C23—H23	119.8
C2—C7—C6	118.6 (2)	C24—C23—H23	119.8
C9—C8—C7	121.21 (18)	C23—C24—C19	121.4 (2)
C9—C8—H8	119.4	C23—C24—H24	119.3
C7—C8—H8	119.4	C19—C24—H24	119.3
C8—C9—N1	121.08 (19)	C30—C25—C26	117.9 (2)
C8—C9—C10	123.64 (18)	C30—C25—C16	119.88 (18)
N1—C9—C10	115.25 (17)	C26—C25—C16	122.21 (18)
C15—C10—C11	117.7 (2)	C27—C26—C25	120.7 (2)
C15—C10—C9	120.50 (19)	C27—C26—H26	119.6
C11—C10—C9	121.8 (2)	C25—C26—H26	119.6
C12—C11—C10	121.2 (2)	C28—C27—C26	120.6 (2)
C12—C11—H11	119.4	C28—C27—H27	119.7
C10—C11—H11	119.4	C26—C27—H27	119.7
C13—C12—C11	119.5 (2)	C29—C28—C27	119.2 (2)
C13—C12—H12	120.3	C29—C28—H28	120.4
C11—C12—H12	120.3	C27—C28—H28	120.4
C12—C13—C14	121.2 (2)	C28—C29—C30	120.6 (2)
C12—C13—Cl1	119.5 (2)	C28—C29—H29	119.7
C14—C13—Cl1	119.4 (2)	C30—C29—H29	119.7
C13—C14—C15	118.9 (2)	C29—C30—C25	120.9 (2)
C13—C14—H14	120.5	C29—C30—H30	119.6
C15—C14—H14	120.5	C25—C30—H30	119.6
C14—C15—C10	121.6 (2)		
			
C16—N2—N3—C18	−0.4 (2)	Cl1—C13—C14—C15	178.42 (18)
C1—N2—N3—C18	−172.66 (16)	C13—C14—C15—C10	0.3 (3)
C9—N1—C1—C2	−0.1 (3)	C11—C10—C15—C14	0.0 (3)
C9—N1—C1—N2	177.32 (15)	C9—C10—C15—C14	179.37 (19)
N3—N2—C1—N1	113.25 (19)	N3—N2—C16—C17	0.5 (2)
C16—N2—C1—N1	−57.8 (2)	C1—N2—C16—C17	172.00 (18)
N3—N2—C1—C2	−69.1 (2)	N3—N2—C16—C25	179.40 (17)
C16—N2—C1—C2	119.8 (2)	C1—N2—C16—C25	−9.0 (3)
N1—C1—C2—C3	176.97 (18)	N2—C16—C17—C18	−0.3 (2)
N2—C1—C2—C3	−0.4 (3)	C25—C16—C17—C18	−179.16 (19)
N1—C1—C2—C7	−0.3 (3)	N2—N3—C18—C17	0.1 (2)
N2—C1—C2—C7	−177.68 (16)	N2—N3—C18—C19	179.74 (16)
C1—C2—C3—C4	−177.35 (18)	C16—C17—C18—N3	0.1 (2)
C7—C2—C3—C4	−0.1 (3)	C16—C17—C18—C19	−179.44 (18)
C2—C3—C4—C5	−0.4 (3)	N3—C18—C19—C20	−7.5 (3)
C3—C4—C5—C6	0.4 (3)	C17—C18—C19—C20	172.01 (19)
C4—C5—C6—C7	0.2 (3)	N3—C18—C19—C24	172.62 (18)
C3—C2—C7—C8	−177.10 (17)	C17—C18—C19—C24	−7.9 (3)
C1—C2—C7—C8	0.4 (3)	C24—C19—C20—C21	0.7 (3)
C3—C2—C7—C6	0.6 (3)	C18—C19—C20—C21	−179.2 (2)
C1—C2—C7—C6	178.07 (17)	C19—C20—C21—C22	−1.1 (3)
C5—C6—C7—C8	176.95 (19)	C20—C21—C22—C23	0.5 (4)
C5—C6—C7—C2	−0.6 (3)	C21—C22—C23—C24	0.5 (4)
C2—C7—C8—C9	0.1 (3)	C22—C23—C24—C19	−0.9 (4)
C6—C7—C8—C9	−177.53 (18)	C20—C19—C24—C23	0.3 (3)
C7—C8—C9—N1	−0.5 (3)	C18—C19—C24—C23	−179.8 (2)
C7—C8—C9—C10	177.49 (17)	C17—C16—C25—C30	−43.5 (3)
C1—N1—C9—C8	0.6 (3)	N2—C16—C25—C30	137.8 (2)
C1—N1—C9—C10	−177.64 (16)	C17—C16—C25—C26	136.1 (2)
C8—C9—C10—C15	−158.10 (19)	N2—C16—C25—C26	−42.5 (3)
N1—C9—C10—C15	20.0 (3)	C30—C25—C26—C27	−2.2 (3)
C8—C9—C10—C11	21.3 (3)	C16—C25—C26—C27	178.2 (2)
N1—C9—C10—C11	−160.58 (18)	C25—C26—C27—C28	0.5 (4)
C15—C10—C11—C12	0.0 (3)	C26—C27—C28—C29	1.2 (4)
C9—C10—C11—C12	−179.4 (2)	C27—C28—C29—C30	−1.2 (4)
C10—C11—C12—C13	−0.3 (4)	C28—C29—C30—C25	−0.5 (4)
C11—C12—C13—C14	0.6 (4)	C26—C25—C30—C29	2.2 (3)
C11—C12—C13—Cl1	−178.40 (18)	C16—C25—C30—C29	−178.2 (2)
C12—C13—C14—C15	−0.5 (4)		
